# Thermally evaporated Cu_2−*x*_Se thin films embedded with PbSe nanoinclusions: a multiphase system for thermoelectric applications

**DOI:** 10.1039/d5ra03803k

**Published:** 2025-07-14

**Authors:** Ammara Shakoor, Sajid Butt, Muhammad Faizan Masoud, Muhammad Umer Iqbal, Muhammad Yasir

**Affiliations:** a Department of Space Science, Institute of Space Technology Islamabad 44000 Pakistan sajid.butt@ist.edu.pk; b Centre for Functional and Surface Functionalized Glass, Alexander Dubcek University of Trencin Slovakia; c Department of Material Science & Engineering, Institute of Space Technology Islamabad 44000 Pakistan

## Abstract

Thermoelectric (TE) devices pave a promising pathway for clean and efficient energy conversion between heat and electricity. Among various TE materials, copper selenide (Cu_2_Se) has attracted significant interest due to its high electrical conductivity and low thermal conductivity offered by its superionic behavior, making it a candidate for high-performance thermoelectric applications. The present study systematically investigates the TE properties of thin film-based nanocomposites comprised of lead (Pb)-doped Cu_(2−*x*)_Se integrated with lead selenide (PbSe) samples. XPS analysis infers the incorporation of Pb and reveals changes in the oxidation states and bonding environments of Cu and Se. Thermoelectric measurements in the temperature range of 300 K to 400 K demonstrate that Pb incorporation influences charge carrier transportation, as inferred alteration in the Seebeck coefficient and electrical conductivity. A slightly overestimated value of the figure-of-merit (*ZT*) has been evaluated by merely relying on the electronic thermal conductivity (*k*_e_) and ignoring the lattice thermal conductivity (*κ*_l_). This work reveals the importance of multiphase compositions in tuning of TE properties and suggests a route for deeper understanding of transport mechanisms in thin film samples.

## Abstract 2

Thermoelectric (TE) devices pave a promising pathway for clean and efficient energy conversion between heat and electricity.

## Graphical abstract

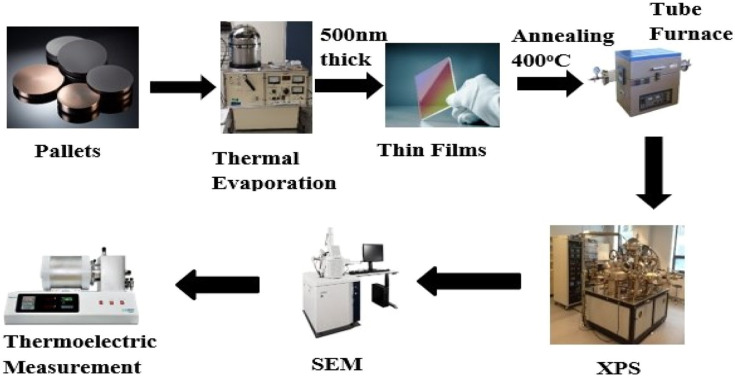

## Introduction

Thermoelectric (TE) devices are gaining immense importance because of their utility in clean and efficient conversion between heat and electricity for sustainable energy solutions. The TE devices, having no moving parts, zero emissions, and without acoustic noise, offer an environmentally friendly solution for electricity generation and solid-state refrigeration by efficiently recycling waste heat being released from industry and automobiles.^1^ They find applications in diverse areas such as automotive seat temperature regulation, industrial waste heat utilization, and low-power wearable or implantable biomedical devices.^2^ The TE performance of a material is determined by its ability to convert heat into electrical energy and *vice versa*. This performance depends on the material's intrinsic properties, typically characterized by the Seebeck coefficient (*S*), electrical conductivity (*σ*), and thermal conductivity (*κ*), as connected by a dimensionless figure of merit: 
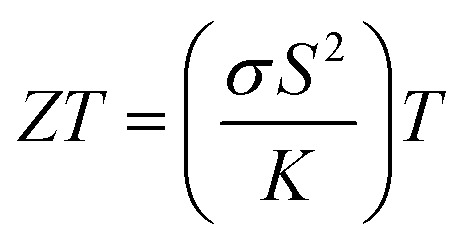
, where *T* the absolute temperature. High-performance (TE) materials are typically characterized by a high power factor (PF) and low thermal conductivity (*κ*), as tailored by tuning of carrier concentration.^3,4^ To enhance *ZT*, several strategies have been adopting including; introducing resonant energy levels or carrier filtering effects,^5^ band structure engineering,^6,7^ optimizing carrier concentration through elemental doping,^8–10^ bulk-nano compositing,^11–13^ grain-texturing,^14–16^ and reduction of lattice thermal conductivity by incorporation of rattling atoms.^17^

In the last two decades, various classes of materials such as chalcogenides,^18,19^ clathrates,^20^ skutterudites^21^ and oxides^7,22^ have undergone extensive investigation in search of efficient TE materials. While oxide-based TE materials traditionally exhibit relatively low (*ZT*) values, which limits their commercial viability, they receive growing attention due to their cost-effectiveness and exceptional thermal stability at elevated temperatures.^23^ Amongst chalcogenides, Cu_(2−*x*)_S has been extensively explored due to its large electrical conductivity and low thermal conductivity offered by superionic behavior.^23,24^ Below 400 K, Cu_(2−*x*)_S crystalizes in numerous crystalline phases such as cubic,^25,26^ tetragonal,^27,28^ orthorhombic,^29,30^ monoclinic,^31,32^ and hexagonal.^33^ Around 400 K, Cu_(2−*x*)_Se undergoes a solid-state phase transition from the low-temperature α-phase to the high-temperature β-phase (cubic only). In both phases, selenium (Se) atoms occupy face-centered cubic (FCC) sites. However, copper (Cu) atoms exhibit distinct behaviors in the α-phase, Cu atoms are well-ordered and confined, whereas in the β-phase, they become kinetically disordered and display superionic conduction behavior.^34,35^ The diverse physical, chemical, and thermal characteristics of copper selenide and its related compounds are caused by variations in composition and crystallography.^36^ Due to the wide range of accessible crystal structures, tunable particle sizes, and compatible band gaps, copper selenide materials exhibit intriguing optoelectronic properties that can be finely tailored for specific applications. Cu_2_Se exhibits a moderate Seebeck coefficient, low thermal and high electrical conductivity. According to the literature, Cu_2_Se synthesized *via* melting followed by spark plasma sintering (SPS) achieves a significant figure of merit (*ZT*) of 1.5 at 1000 K, demonstrating its potential for high-temperature thermoelectric applications.^37^ Doping tellurium (Te) at the selenium site enabled Cu_2_Se to have a higher *ZT* value of 1.9 at 873 K.^38^ Using S-doped Cu_2_Se, (*ZT*) was further improved up to 2.0 at 1000 K.^39^ Currently, the Al-doped Cu_2_Se bulk has the greatest (*ZT*) value, 2.62 at 1029 K.^40^ Extensive research has been conducted on doping Cu_2_Se to enhance its thermoelectric (TE) performance, with numerous studies confirming that dopants significantly influence the material's (TE) properties. For instance, incorporating aluminum (Al) can modify the microstructure of Cu_2_Se, thereby affecting its thermoelectric behavior. At 1029 K, the highest (*ZT*) value recorded for Al-doped Cu_2_Se bulk materials is 2.62. A variety of dopants, spanning across all major groups as well as several transition elements, have been explored for doping Cu_2_Se. Qiujun Hu *et al.* synthesized Li-doped Cu_1.98_Li_0.02_Se bulk materials using hydrothermal synthesis followed by hot pressing, achieving a high (*ZT*) value of 2.14 at 973 K.^41^ Zhao *et al.* have carried out extensive studies on Cu_2_Se doped by a range of carbon resources, including graphite (G), carbon nanotubes (CNT), super P (SP), and hard carbon (H). The greatest (*ZT*) value of 2.4 at 850 K was obtained by 0.3 weight percent carbon fiber-doped Cu_2_Se. Melt quenching and solid-state reactions are used to create the composites.^42,43^ Moreover, Zhu *et al.* produced a Pb-doped Cu_2_Se material with the same layered structure.^44^ They discovered in their experiments that the thermal conductivity decreases with the lamellae width and grain boundaries effectively disperse phonons and carriers.

Although bulk Cu_2_Se exhibits superior thermoelectric (TE) performance compared to its thin film counterparts, but Cu_2_Se thin films offer distinct advantages like reducing materials consumption. In addition to reducing material consumption and associated costs, thin films are particularly suitable for device miniaturization and integration into microscale systems. Various deposition techniques for Cu_(2−*x*)_Se thin films have been reported in the literature, including solution,^39,45^ electrodeposition,^46^ pulsed laser deposition,^47^ ion beam sputtering deposition,^48^ reactive evaporation,^49^ and pulsed/magnetron sputtering.^50^

In this work, the thermoelectric transport properties of Pb-doped Cu_(2−*x*)_Se thin films have been systematically explored and explained with the help of detailed structural characterizations. Comparing vacuum thermal evaporation to other thin film deposition techniques, it offers several benefits, including consistent film thickness, good compaction, and adhesion to glass substrate. It is also readily available.

## Experimentation

### Preparation of thin films

**Fig. 1:**
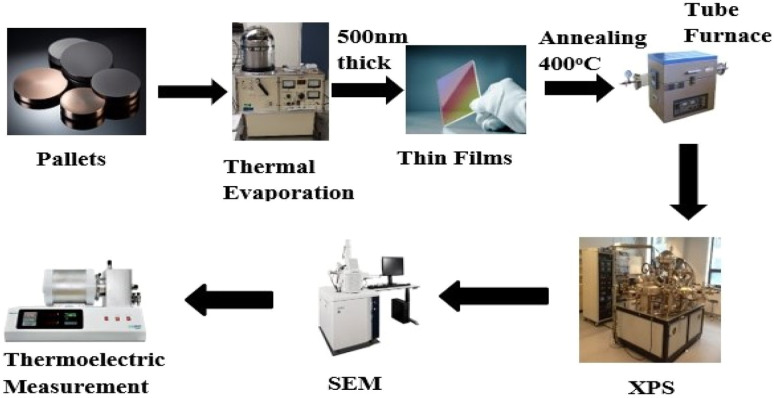
Schematic diagram of revealing the fabrication and structural and thermoelectric characterization of pristine and Pb-doped Cu_2−*x*_Se thin films.

The complete synthesis and characterization procedure is illustrated in Fig. 1. A series of thin films, including pristine Cu_2_Se and lead-doped variants Cu_2_Se_0.95_Pb_0.05_ (Pb-5%), Cu_2_Se_0.9_Pb_0.1_ (Pb-10%), Cu_2_Se_0.75_Pb_0.25_ (Pb-25%), Cu_2_Se_0.5_Pb_0.5_ (Pb-50%), and Cu_2_Se_0.25_Pb_0.75_ (Pb-75%) were synthesized *via* thermal evaporation technique. For all the series, films having a thickness of 500 nm were deposited over a pre-cleaned glass substrate, using Nanovak NVBJ-300 TH thermal evaporator equipped with a tungsten boat. The chamber was evacuated to a pressure of 5.0 × 10^−6^ mbar before deposition. A current in the range of 90–100 A was applied to achieve a stable deposition rate of 0.5 Å s^−1^. The as-deposited films were annealed at 400 °C for 1 hour to in an inert atmosphere.

### Characterization

X-ray diffraction (XRD) patterns were obtained using BRUKER diffractometer (D2 PHASER) equipped with Cu-Kα radiations (*λ* ∼1.54 Å). XPS (X-ray Photoelectron Spectroscopy), a surface-sensitive analytical technique, was used to determine chemical states of all the elements. The high-resolution spectra of Cu2p, Se3d, and Pb4f were deconvoluted using XPSPEAK41 software. A Scanning Electron Microscopy (SEM) model (ZEISS Gemini 300-8202017309) was used to study surface morphology and phase distribution of all the pristine and Pb-doped Cu_2_Se-based thin films using secondary electrons (SE) and backscattered electrons (BSE), respectively. The particle size distribution was estimated from SEM images using IMAGEJ software. The qualitative and quantitative elemental composition was determined using energy dispersive X-ray spectroscopy (EDS), as equipped with SEM. The electrical conductivity and Seebeck coefficient were measured for the α-phase over a temperature range of 300–400 K, using the four-probe geometry with a Thermoelectric Parameter Testing System (Joule-Yacht-Namicro-3L).

## Results and discussion

**Fig. 2:**
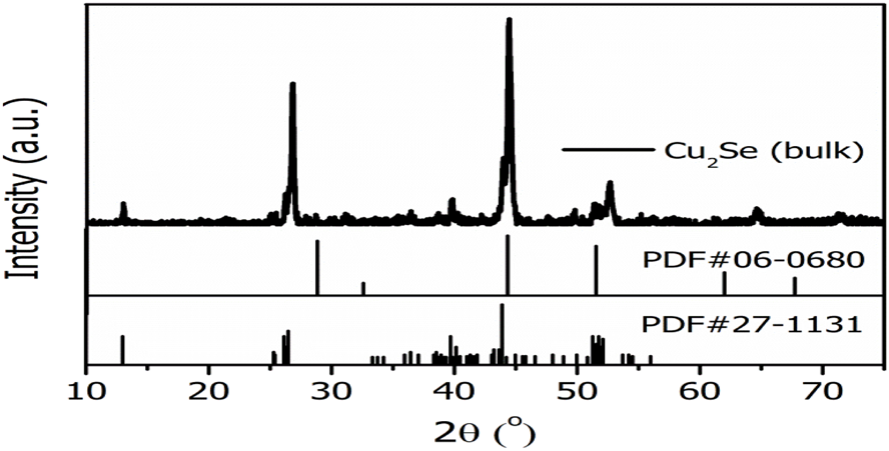
Room temperature XRD pattern of the bulk Cu_(2−*x*)_Se sample employed for thin film deposition.

Fig. 2 shows the X-ray diffraction (XRD) patterns of powdered Cu_(2−*x*)_Se recorded at room temperature. The powder sample exhibits well-defined diffraction peaks corresponding to monoclinic and cubic polymorphs, as indexed by PDF# 27-1131 and PDF# PDF#06-0680, respectively.^23,51^ In contrast, the XRD patterns of the Cu_(2−*x*)_Se thin films display no distinct diffraction peaks. Instead, a broad amorphous hump was observed, which originates from the glass substrate on which the films were deposited. As a result, structural analysis of the thin films using XRD was not feasible. Therefore, X-ray photoelectron spectroscopy (XPS) was employed to investigate the composition and chemical states of the elements present in the films.

**Fig. 3:**
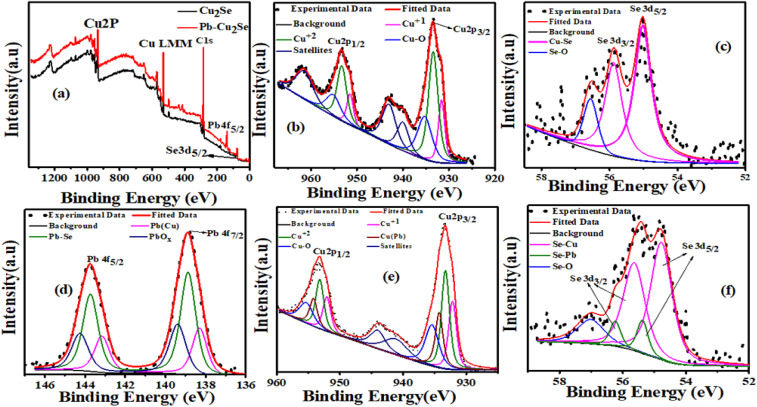
XPS analysis of Cu_2_Se and Pb-5% doped Cu_(2−*x*)_Se thin films, (a) full survey spectra showing elemental peaks. (b, e) Cu 2p spectra, (c, f) Se 3d spectra (d) Pb 4f spectrum for pristine and Pb-doped Cu_(2−*x*)_Se thin films.

The XPS full survey spectra of pristine Cu_(2−*x*)_Se and Pb-5% doped Cu_(2−*x*)_Se thin films are shown in Fig. 3(a). The presence of Cu, Se, and Pb is confirmed by the respective core-level binding energies. C 1s peak located near 284.8 eV can be seen clearly, which serves the purpose of reference for charge correction.^52^Fig. 3(b) presents the high-resolution Cu 2p spectrum. The peaks corresponding to Cu^+^ and Cu^2+^ oxidation states confirm the mixed valence nature of copper, which is characteristic of the non-stoichiometric phase and superionic behavior of Cu_(2−*x*)_Se.^12^ The slight shift in Cu^1+^ binding energy suggests changes in the local bonding environment, which is in good agreement with that of the previously reported data on pure and doped Cu_(2−*x*)_Se.^23,51,53^ However, the Cu–O component indicates surface oxidation or interaction with residual oxygen. The Se 3d core-level spectrum in Fig. 3(c) shows two well-resolved peaks at ∼54.2 eV and ∼55.2 eV, assigned to Se 3d_5/2_ and Se 3d_3/2_ which are spin–orbit split components, respectively. Peaks attributed to Se–Cu bonding confirm the selenide phase, while additional Se–O components suggest partial surface oxidation of selenium.

Fig. 3(d) displays the Pb 4f spectrum, where Pb 4f_7/2_ and Pb 4f_5/2_ peaks confirm the presence of Pb^2+^ species. The deconvolution reveals Pb–Se bonds, indicating the formation of PbSe, as well as minor contributions from Pb–O, likely due to surface oxidation. The appearance of Pb–Cu interactions indicates the partial substitution of Pb at the Cu site. In the Cu 2p spectrum for the Pb-doped sample, Fig. 3(e), changes in peak intensity and satellite structure are observed. A reduction in Cu^2+^ satellite intensity may indicate partial reduction to Cu^+^, possibly influenced by Pb doping as a result of charge redistribution. The presence of Cu–Pb bonds further supports electronic interactions between Cu and Pb, indicating the partial substitution of Pb at Cu-site. Fig. 3(f) shows the Se 3d spectrum of the Pb-doped sample. Se–Pb bonding suggests the formation of PbSe phases. A noticeable decrease in the Se–Cu component and the persistence of Se–O indicates the redistribution of selenium atoms for the formation of PbSe phase. A slight shift in Se 3d binding energies compared to that of the pristine Cu_(2−*x*)_Se sample may reflect electronic structure modification due to Pb incorporation.

**Fig. 4:**
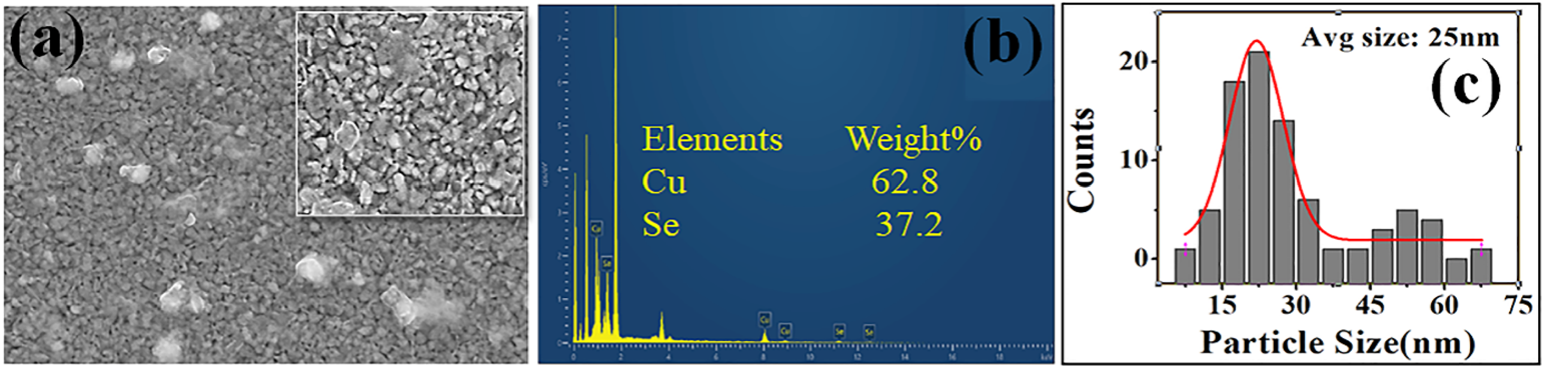
Surface morphology and compositional analysis of Cu_2−*x*_Se thin film. (a) FESEM image showing uniform nanoparticle distribution, with an image in the inset showing clear particle size with a range nearly equal to 100 nm. (b) EDS spectrum confirming the presence of Cu and Se elements in pristine Cu_2−*x*_Se thin films. (c) Average particle size ∼25 nm.

Fig. 4(a) presents the SEM image, revealing a relatively uniform distribution of grains with some degree of agglomeration and minor voids. The magnified image indicates a nanocrystalline structure with a clear particle size and shape, as shown in the inset of Fig. 4(a). The particle size distribution, showing an average particle size of approximately 25 nm, in Fig. 4(c) is consistent with a fine-grained nanostructure that can enhance phonon scattering in thermoelectric applications. EDS analysis confirms the presence of Cu and Se elements in the pristine film, affirming the successful deposition of the desired phase, as shown in Fig. 4(b).

**Fig. 5:**
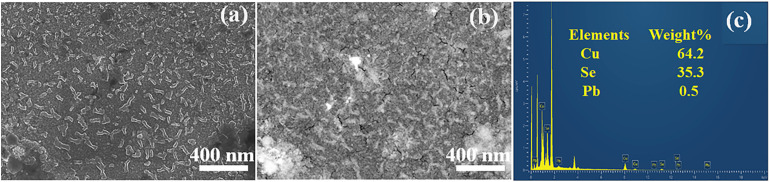
(a) Secondary electron (SE) image, (b) backscattered electron (BSE) image, and (c) elemental composition (EDS)of Cu_2_Se_0.95_Pb_0.05_ (Pb-5%) sample.

Fig. 5(a) presents the SEM and elemental concentration analysis of Pb-doped 5% in Cu_2_Se thin films. The secondary electron (SE) SEM image reveals the surface morphology, showing a granular structure. (Pb) particles appear prominently on the surface, while Cu_2_Se particles are distributed in the background. In Fig. 5(b), the backscattered electron (BSD) image highlights compositional contrast; brighter regions represent the PbSe inclusions, while darker regions indicate areas containing Cu_(2−*x*)_Se matrix. The EDS spectrum confirms the presence of Cu, Se, and Pb in the Pb-doped sample, as shown in Fig. 5(c). The combined XPS, SEM and EDS results confirm the formation of a multiphase system consisting of Cu_(2−*x*)_Se matrix embedded with PbSe inclusions.

**Fig. 6:**
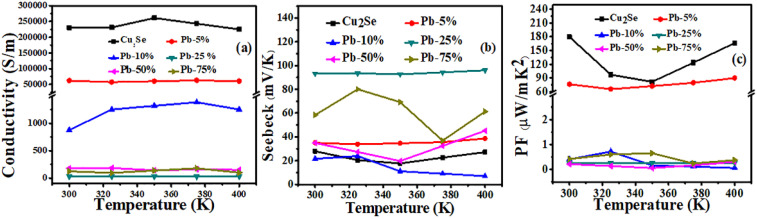
(a) Shows the temperature-dependent electric conductivity, (b) Seebeck coefficients, and (c) power factor (PF) of all the series of pristine and Pb-doped Cu_(2−*x*)_Se thin films.

The temperature dependent electrical conductivity (*σ*) and Seebeck coefficient (*S*) were measured in the temperature range of 300 K to 400 K.

(TE) temperature-dependent parameters, electrical conductivity, Seebeck coefficient, and power factor (PF) for all samples are summarized in Fig. 6. Among the doped series, the Pb-5% sample exhibits the highest electrical conductivity, approximately 5.0 × 10^4^ S m^−1^, whereas the pristine Cu_2_Se sample maintains the highest overall conductivity, as illustrated in Fig. 6(a). It can be seen that the electrical conductivity of all the doped series has decreased as compared to the pristine Cu_(2−*x*)_Se sample. This decrease is associated with the incorporation of a small amount of Pb into the Cu_(2−*x*)_Se lattice introduces point defects that act as scattering centers, thereby reducing the electrical conductivity across all doped series, *i.e.*, 10%, 50%, and 75%. Furthermore, excess Pb leads to the formation of a secondary PbSe phase, as confirmed by XPS and SEM analyses. This secondary phase further impedes carrier transport, contributing to an additional decline in electrical conductivity.

The activation energies of all the series of pure and Pb-doped samples were calculated using the Arrhenius equation as given in eqn (1).1
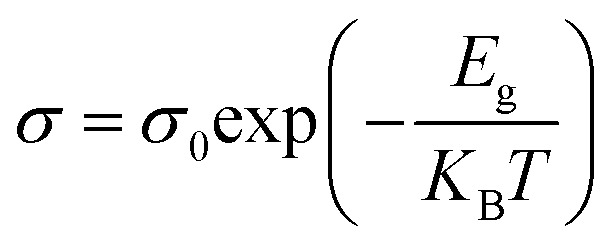
where, *σ*, *T*, *K*_B_, and *E*_g_ represent the electrical conductivity, temperature, Boltzmann constant and activation energy.^52^

The *E*_g_ values for all the series are listed in Table 1, and variations in activation energy are observed across the doped samples, likely due to fluctuations in electrical conductivity. Pb-10% doped sample shows the highest activation energy among all the samples, while 5% doped sample shows the lowest energy due to the highest electrical conductivity among all samples. Pb-25% and 50% doped samples show low energies along with low conductivities that can be associated with defects and multi-phases.

**Table 1:** Activation energies of pristine Cu_(2−*x*)_Se and Pb-doped samples Cu_(2−*x*)_Se

Samples	*E*_g_ (eV)
Pure Cu_2_Se	0.04
Pb-5%	0.03
Pb-10%	0.09
Pb-25%	0.03
Pb-50%	0.01
Pb-75%	0.08

A consistently positive Seebeck coefficient across the investigated temperature range confirms holes are the dominant charge transport in both pristine and Pb-doped Cu_2_Se thin films, as shown in Fig. 6(b). The Seebeck coefficient trends, shown in Fig. 6(b), reveal a complex dependence on Pb doping. The pure Cu_2_Se thin film demonstrates a modest Seebeck coefficient (∼35 μV K^−1^), consistent across the temperature range. Interestingly, the 25% Pb-doped sample displays the highest Seebeck coefficient, 93.1 μV K^−1^, suggesting a favorable balance between carrier concentration and energy-dependent carrier filtering.^54^ However, doping beyond Pb-25% results in reduced Seebeck values, likely due to reduced carrier mobility and altered band structure.

Despite the enhancement in Seebeck values, the dominant suppression of electrical conductivity leads to an overall decline in the power factor with higher doping concentrations. The pristine Cu_2_Se film records the maximum (PF) value of 179.7 μW mK^−2^, while among the doped samples, the Pb-5% film exhibits the highest power factor of 89.9 μW mK^−2^ at the same temperature of 400 K as shown in Fig. 6(c). This decline reaffirms that excessive Pb doping impairs thermoelectric performance due to decreased electrical conductivity. These observations align with prior studies indicating that moderate doping can enhance carrier energy filtering, while excessive dopant levels introduce defect scattering and structural disorder.^55^

**Fig. 7:**
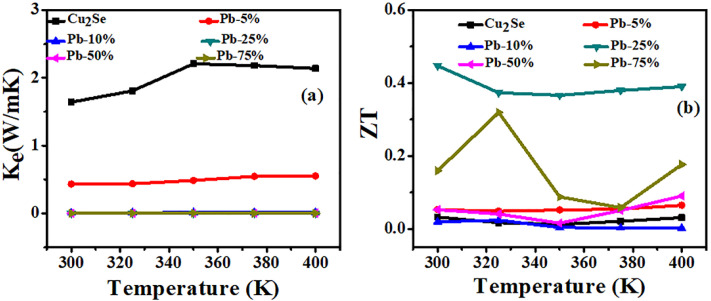
Temperature-dependent (a) electronic thermal conductivity (*K*_e_) and (b) figure of merit (*ZT*) of all the series of pristine and Pb-doped Cu_(2−*x*)_Se films.

The electronic thermal conductivity (*K*_e_) of the entire series of films, as shown in Fig. 7(a), has been calculated using the Wiedemann–Franz Law as given in eqn (2);

2 *k*_e_ = *L*_o_*σT*

(*k*_e_) of pure thin film is increasing with temperature and stable above 340 K, indicating good transport properties. Doping with Pb-5% reduces (*k*_e_) as compared to pure thin films. The highest value of (*k*_e_) is 2.2 W mK^−1^. This reduction may be because of increasing charge carriers scattering at the intergranular interfaces and defect sites, shown by doping. Overall, as doping concentration increases, electronic thermal conductivity decreases. The estimated *ZT* values in the temperature range of 300 K to 400 K were calculated by considering only the electronic thermal conductivity (*κ*_e_), while neglecting the lattice thermal conductivity (*κ*_l_). As shown in Fig. 7(b), the obtained (*ZT*) values are slightly overestimated due to the exclusion of lattice thermal conductivity (*κ*_l_) in the calculations. Nevertheless, they provide valuable insight into the changes in transport mechanisms induced by Pb doping in the films. Among all the Cu_2_Se thin films, the sample doped with 25% Pb exhibits the highest (*ZT*) value, primarily due to its lowest electronic thermal conductivity (*κ*_e_). The maximum (*ZT*) value observed is 0.4 at 300 K, which, while overestimated, still highlights the promising potential of this composition for thermoelectric applications.

## Conclusion

In this study, the thermoelectric properties of Pb-doped Cu_(2−*x*)_Se thin films synthesized *via* thermal evaporation were systematically investigated across a temperature range of 300 K–400 K. The comprehensive structural characterization confirmed the partial incorporation of Pb into Cu_(2−*x*)_Se lattice along with the formation of PbSe secondary phase. The thermoelectric measurements showed that Pb doping alters the transportation mechanism, resulting in tunable electrical conductivity and Seebeck coefficient values. The consistently positive Seebeck coefficients confirmed p-type conduction dominated by holes, with the Pb-5% doped sample exhibiting the highest electrical conductivity among the doped variants. Despite the overestimation of (*ZT*) 0.4 due to the exclusion of lattice thermal conductivity, it suggests that controlled Pb incorporation can improve the thermoelectric performance of Cu_2−*x*_Se thin films. These findings demonstrate the potential of Pb-doped Cu_(2−*x*)_Se thin films as a viable candidate for integration into microscale thermoelectric devices, and greatly help in understanding the transportation mechanism in Cu_(2−*x*)_Se and related materials.

## Conflicts of interest

All authors declare that there are no conflicts of interest.

## Data Availability

Data will be provided upon request.
